# Identification of hepatic metastasis from an unrevealed adenoid cystic carcinoma by PET/CT: A case report

**DOI:** 10.1097/MD.0000000000039769

**Published:** 2024-09-20

**Authors:** Ze Liang, Yi Ding, He Sui, Mei Wu, Yongmin Jin, Weibo Wen

**Affiliations:** a Department of Nuclear Medicine, Yanbian University Hospital, Yanji, China; b Department of Radiology, Yanbian University Hospital, Yanji, China; c Department of General Practice, Yanbian University Hospital, Yanji, China; d Department of Dalian Rehabilitation and Convalescence Center, China; e Department of Oncology, Yanbian University Hospital, Yanji, China.

**Keywords:** ^18^F-FDG PET/CT, adenoid cystic carcinoma, liver metastasis, unknown primary

## Abstract

**Rationale:**

Adenoid cystic carcinoma is a rare malignant tumor of the salivary glands, with few reports of metastasis to the liver in the literature. We present a case where an isolated hepatic lesion of adenoid cystic carcinoma was identified using ^18^F-fluorodeoxyglucose positron emission tomography/computed tomography (^18^F-FDG PET/CT).

**Patient concerns:**

A 76-year-old male experienced abdominal pain and underwent an enhanced CT scan and magnetic resonance imaging, which revealed a liver mass. Subsequent ^18^F-FDG PET/CT identified hypermetabolic lesions in both the left and right lobes of the liver, suggesting malignancy, with no other abnormalities detected.

**Diagnoses:**

A liver biopsy confirmed the diagnosis of adenoid cystic carcinoma.

**Interventions:**

No intervention.

**Outcomes:**

Following confirmation of the diagnosis, the patient chose to discontinue treatment and was discharged.

**Lessons:**

Hepatic metastasis from adenoid cystic carcinoma may be detected before the identification of the primary lesion. ^18^F-FDG PET/CT plays a critical role in differentiating benign from malignant liver tumors, selecting potential biopsy sites, and assessing the extent of metastatic disease.

## 1. Introduction

Adenoid cystic carcinoma (ACC) is an uncommon, idiopathic cancer mainly impacting women, with a primary occurrence in the head and neck areas.^[1]^ It is marked by gradual growth, invasive behavior, and a strong likelihood of local recurrence and distant metastasis.^[2]^ The lungs and bones are the most frequent sites of metastasis for ACC.^[1]^ However, in rare cases, ACC can present with hepatic metastasis. ACC has unique pathological features, treatment needs, and prognosis. ACC may produce excessive hormones and cause specific symptoms, which require special treatment and management strategies, such as surgery, drug, and hormone therapy.^[3]^ Since ACC typically carries a poor outlook, the early diagnosis of metastatic ACC lesions is essential for the provision of early effective interventions. ^18^F-fluorodeoxyglucose positron emission tomography/computed tomography (^18^F-FDG PET/CT) represents the forefront of molecular imaging technology in clinical use and can significantly enhance the detection rate of pathological lesions particularly primary cancerous lesions of unknown origin.^[4]^ In this case study, we report a patient diagnosed with biopsy-confirmed hepatic ACC lesions of an unknown primary origin.

## 2. Case report

**Figure 1.**
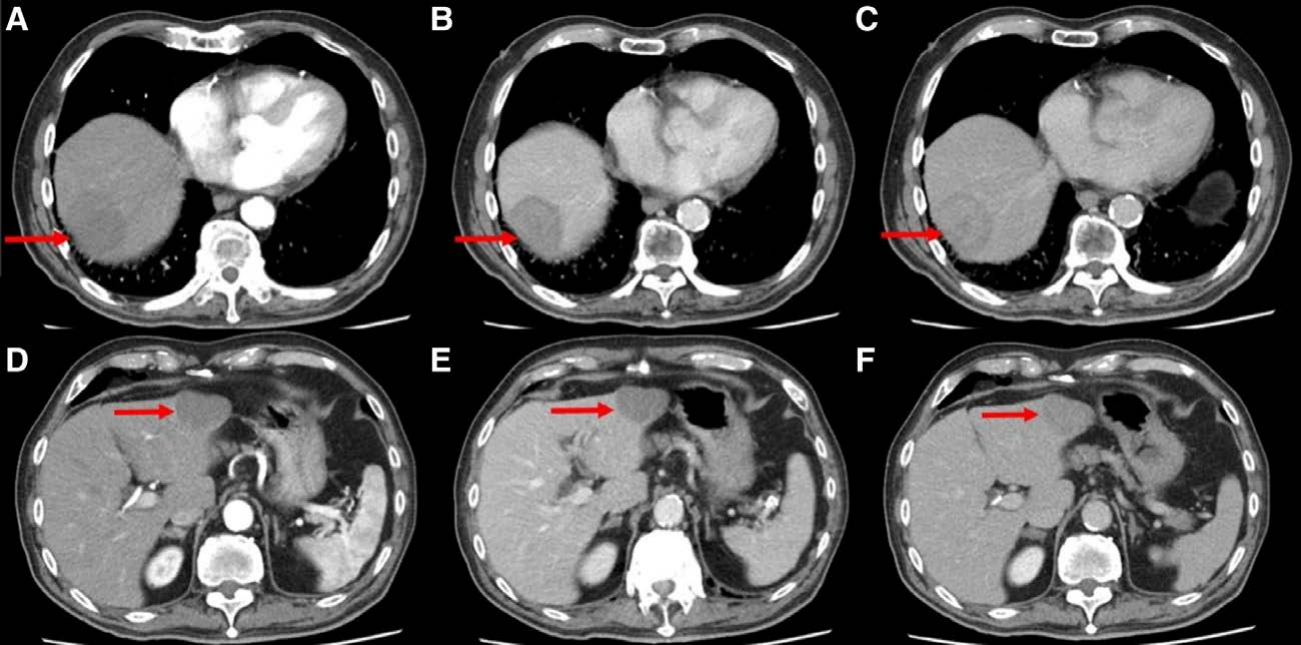
Enhanced abdominal CT images showing lesions in both the right and left lobes of the liver lobes (indicated by red arrows). Images (A–D) demonstrate peripheral enhancement during the arterial phase, while images (C–F) show delayed enhancement. CT = computed tomography.

**Figure 2.**
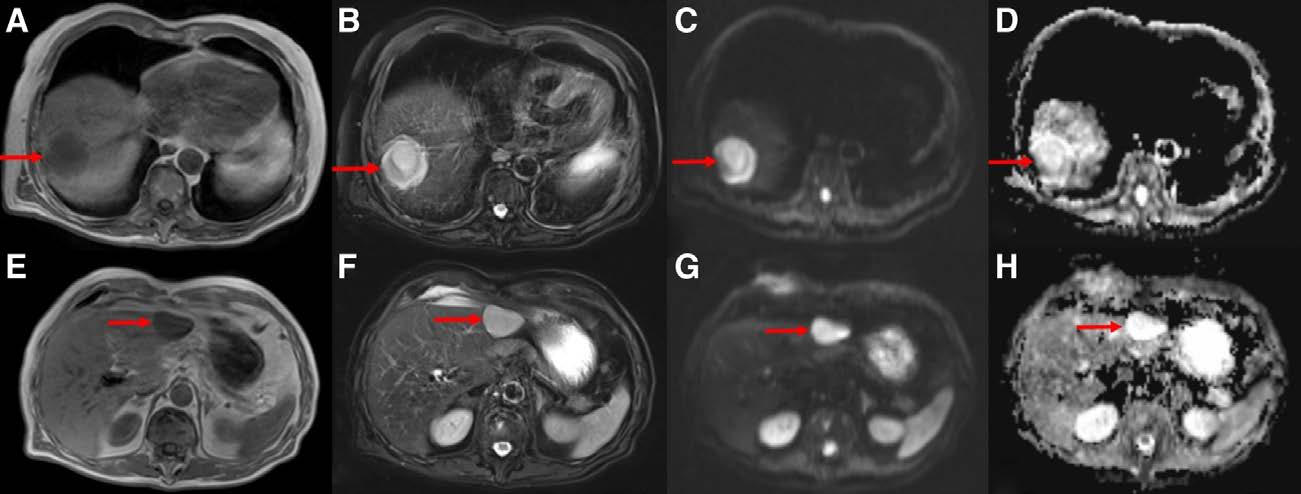
Liver MRI without contrast showing lesions in both the right and left liver lobes. T1WI images (A/E), T2WI image (B/F), DWI image (C/G), ADC image (D/H). ADC = apparent diffusion coefficient, DWI = diffusion-weighted imaging, MRI = magnetic resonance imaging, T1WI = T1-weighted imaging, T2WI = T2-weighted imaging.

**Figure 3.**
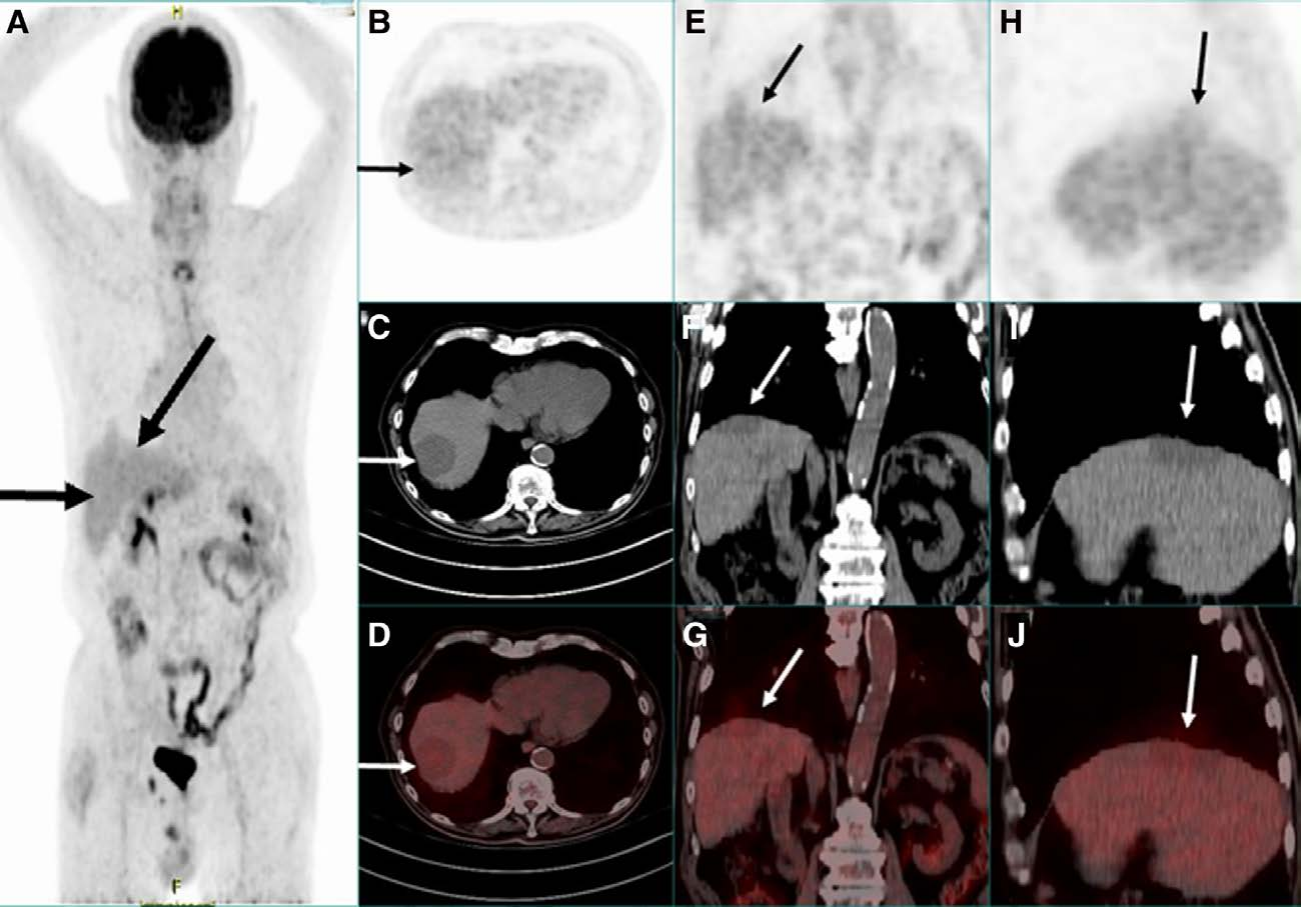
MIP PET/CT images are displayed. Image (A) reveals lesions within the left and right parts of the liver (marked by black arrows). Images (B) through (D) present axial views of the PET (B), CT (C), and fused PET/CT (D) (marked by black and white arrows). Images (E) through (G) depict the coronal views of the PET (E), CT (F), and fused PET/CT (G) (marked by black and white arrows). Images (H) through (J) illustrate the sagittal views of the PET (H), CT (I), and fused PET/CT (J) (marked by black and white arrows). The PET/CT images display round, slightly hypodense lesions, with the largest measuring up to 5.0 cm in maximum length. The maximum standardized uptake value (SUVmax) for these lesions was recorded at 4.4. MIP = maximum intensity projection, PET/CT = positron emission tomography/computed tomography.

An elderly male patient experienced abdominal pain and underwent an enhanced CT scan, which revealed round, low-density lesions across both the right and left liver lobes. The largest lesion measured approximately 5.0 cm in diameter. The lesions exhibited slight enhancement at the margins during the arterial phase and mild delayed enhancement (Fig. 1). Subsequent magnetic resonance imaging (MRI) of the liver showed that these masses displayed reduced signal intensity on T1-weighted imaging and elevated signal intensity displaying low signal septations on T2WI diffusion-weighted imaging (DWI)showed elevated signal intensity, while the apparent diffusion coefficient (ADC) showed a reduced signal intensity within the lesion (Fig. 2). Further assessment with ^18^F-FDG PET/CT indicated elevated metabolic activity in these liver lesions, indicative of malignancy, with no other abnormalities identified throughout the body (Fig. 3).

**Figure 4.**
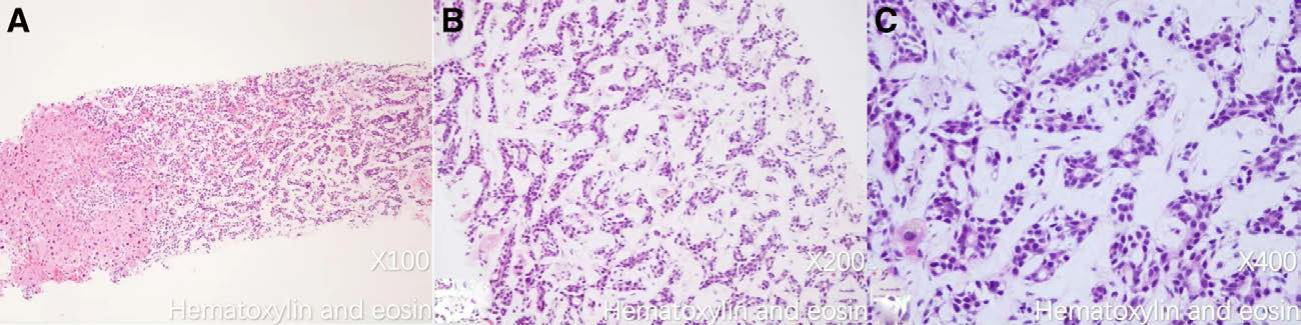
H&E pathology images at various magnifications (A ×100; B ×200; C ×400). At the tumor-liver interface, basaloid cells arranged in trabecular and tubular structures were noted.

**Figure 5.**
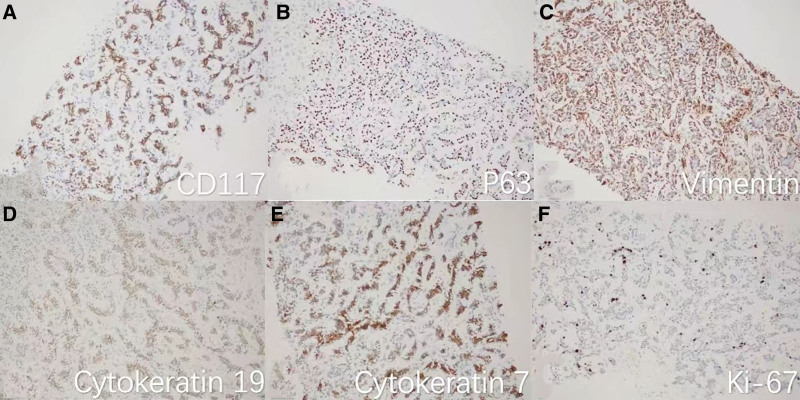
Immunohistochemical pathology images (×200) showing positive staining for CD117 (A), P63 (B), vimentin (C), cytokeratin 19 (D), cytokeratin 7 (E), and 30% Kiel clone 67 (Ki-67) (F). H&E = hematoxylin and eosin.

The patient did not have any history of malignancy or familial history of cancer. Physical examination upon admission showed jaundice in the skin and sclera but no other significant findings. Laboratory tests revealed an aspartate aminotransferase of 73 U/L (normal range: 0–40 U/L) and alanine aminotransferase of 246 U/L (normal range: 0–40 U/L). All biomarkers, including the carcinoembryonic antigen, alpha-fetoprotein, cancer antigen 125, cancer antigen 153, cancer antigen 199, and cancer antigen 72-4, were within normal limits. A CT-guided biopsy of the high FDG uptake liver lesions was conducted, and histological analysis using hematoxylin and eosin staining as well as immunohistochemical staining (Figs. 4 and 5) confirmed the presence of metastatic ACC. Following the definitive diagnosis, the patient declined surgical treatment and opted for conservative management before electing to be discharged.

## 3. Discussion

ACC is a malignancy predominantly affecting women, characterized by a high propensity for late metastasis, often occurring more than 20 years after the primary tumor is excised.^[5]^ This pattern of delayed metastasis was first described by van der Wal et al.^[6]^ Although surgical resection with clean margins typically allows for the histopathological identification of remote ACC metastases, these metastases are often too minute for detection by clinical examination or conventional imaging techniques.^[7]^ Early identification and precise staging are essential for the provision of timely effective interventions. However, traditional diagnostic techniques like CT and MRI often lack the specificity needed for detecting isolated metastatic ACC. Studies have shown that ^18^F-FDG PET/CT has greater sensitivity compared to CT and MRI in the detection of small carcinomas of unknown primary origin.^[8]^ Moreover, ^18^F-FDG PET/CT provides a sensitive instrument for identifying and evaluating the scope of distant metastases when the histopathology is inconclusive.

PET/CT is an imaging technique that makes use of radioactive pharmaceutical isotopes to capture metabolic information within lesions. Compared to normal tissue, cancer cells tend to have a high ^18^F-FDG uptake as a result of increased vascularity, cellular density, glycolysis, and cell reproduction.^[9]^ Maximum Standardized Uptake Value（SUVmax） quantifies the concentration of a radiotracer within a tissue, normalized to the injected dose of the radiotracer and the patient’s body weight. The SUVmax value under normal conditions should be <2.0.^[10]^ Consequently, this measure is frequently utilized to differentiate between benign and metastatic lesions. Research indicates that PET/CT imaging demonstrates substantial diagnostic precision for various cancers of unknown primary, such as metastatic cancers in the head and neck,^[11]^ cervical region,^[12]^ and bones.^[13]^ Nevertheless, there are scant reports on the diagnostic efficacy of ^18^F-FDG PET/CT in liver metastases from cancers of unknown primary origins, especially ACCs.

In this report, we describe an uncommon case of a patient identified with hepatic ACC of unknown primary origin. The enhanced abdominal CT scan of this patient revealed circular, low-density lesions in the liver featuring slight arterial enhancement and mild delayed enhancement. The liver MRI indicated that the lesions had a low T1 and ADC signal, a high uneven T2 signal, and a high DWI signal. The MRI result indicated the presence of benign lesions such as hepatic hemangiomas^[14]^ or focal nodular hyperplasia.^[15]^ However, the high SUVmax values identified by the ^18^F-FDG PET/CT indicated malignancy. Following these observations, a biopsy was conducted the location of the ^18^F-FDG uptake was used to guide the tissue biopsy. The whole-body scan ^18^F-FDG PET/CT did not reveal other metastatic sites. Unfortunately, the ^18^F-FDG PET/CT scan did not successfully locate the primary lesion thus highlighting the limitations of this technology in detecting ACC lesions obscured by normal FDG uptake and those with relatively low metabolism or nonsignificant FDG uptake.

11 carbon-choline (^11^C-choline) is a novel radiotracer that could offer an alternative to ^18^F-FDG. This radiotracer offers a clearer visualization of lesions and their margins. It is more accurate for tumor biological target positioning and T staging. Second, the uptake concentration of ^11^C-choline in the inflammatory site is not high, which can distinguish part of the inflammation from the tumor, and to a certain extent, it can compensate for the lack of FDG. In addition, ^11^C-choline imaging is more sensitive than ^18^F-FDG imaging, and its SUV threshold is lower than ^18^F-FDG.^[16–18]^

Since ^11^C-choline can provide high-contrast images, it could potentially be used to identify ACC within the head and neck obscured by normal physiological ^18^F-FDG uptake. Consequently, ^11^C-choline PET/CT might surpass ^18^F-FDG PET/CT in diagnosing the primary lesion of hepatic ACC of unknown origin. Nonetheless, additional extensive prospective studies are needed to verify this hypothesis.

## 4. Conclusion

In summary, this report showed a significant value of ^18^F-FDG PET/CT in detecting ACC metastases with an unknown primary lesion. We, therefore, recommend the application of this imaging technique to detect metastasis from ACC at an early stage and hence improve treatment outcomes.

## Acknowledgments

The authors would like to thank TopEdit (www.topeditsci.com) for the English language editing of this manuscript.

## Author contributions

**Conceptualization:** Ze Liang, Yongmin Jin, Weibo Wen.

**Writing**—**original draft:** Ze Liang, Yi Ding.

**Writing**—**review & editing:** Ze Liang, Weibo Wen.

**Data curation:** Yi Ding, He Sui, Mei Wu, Yongmin Jin, Weibo Wen.

**Supervision:** Yongmin Jin, Weibo Wen.

**Validation:** Weibo Wen.
